# Why all blood donations should be tested for hepatitis E virus (HEV)

**DOI:** 10.1186/s12879-019-4190-1

**Published:** 2019-06-20

**Authors:** Joachim Denner, Sven Pischke, Eike Steinmann, Johannes Blümel, Dieter Glebe

**Affiliations:** 1 Robert Koch Institute, Nordufer 20, 13353 Berlin, Germany; 2 1. Medizinische Klinik, Universitätsklinikum Hamburg-Eppendorf, Martinistrasse 52, 20246 Hamburg, Germany; 3 Ruhr-Universität Bochum, Universitätsstraße 150, 44801 Bochum, Germany; 4 Paul-Ehrlich-Institut, Paul-Ehrlich-Straße 51-59, 63225 Langen, Germany; 5 Institute of Medical Virology, National Reference Centre for Hepatitis B and D Viruses, German Center for Infection Research (DZIF), Schubertstr. 81, Justus-Liebig-Universität Giessen, 35392 Giessen, Germany

**Keywords:** Hepatitis E virus, Blood donations, Blood transfusion, Safety, Blood testing

## Abstract

**Background:**

Hepatitis E is a liver disease caused by a small RNA virus known as hepatitis E virus (HEV). Four major genotypes infect humans, of which genotype 1 and 2 (HEV-1, HEV-2) are endemic mainly in Asia and responsible for waterborne epidemics. HEV-3 and HEV-4 are widely distributed in pigs and can be transmitted to humans mainly by undercooked meat, and contact with pigs. HEV-3 is the main genotype in industrialised countries with moderate climate conditions and object of this debate.

**Main text:**

Whereas an HEV-3 infection in healthy humans is mostly asymptomatic, HEV-3 can induce chronic infection in immunocompromised individuals and acute-on-chronic liver failure (ACLF) in patients with underlying liver diseases. The number of reported cases of HEV-infections in industrialised nations increased significantly in the last years. Since HEV-3 has been transmitted by blood transfusion to other humans, testing of blood donors has been introduced or introduction is being discussed in some industrialised countries. In this article we summarise the arguments in favour of testing all blood donations for HEV-3.

**Conclusion:**

The number of HEV infection in the population and the possibility of HEV transmission by blood transfusion are increasing. Transmission by blood transfusion can be dangerous for the recipients considering their immunosuppressive status, underlying disease or other circumstances requiring blood transfusion. This argues in favour of testing all blood donations for HEV-3 to prevent transmission.

## Background

Blood transfusion is a lifesaving procedure to replace blood cells or plasma lost through severe bleeding, e.g., during surgery when blood loss occurs. It is also used in anemic patients as an essential part of treatment. In 2014, nearly 3.8 million units of packed red blood cells, 485,000 thrombocyte or platelet concentrates, and 796,000 units plasma were used for transfusions in Germany [1].

In order to avoid transmission of microorganisms from the donor to the recipient, strong regulations are in force. All donations in Germany must be tested for antibodies against human immunodeficiency virus 1 (HIV-1) and HIV-2, against human hepatitis C virus (HCV), against human hepatitis B core antigen (HBcAg), as well as for human hepatitis B surface antigen (HBsAg) and for the genomes of HCV and HIV using nucleic acid testing (NAT) [2]. Thus, transfusion-associated transmissions of these viruses are rare [3]. Also transmission of other viruses including West Nile virus (WNV), human T cell lymphotropic virus (HTLV), rabies virus as well as of parasites (mainly malaria) and bacteria has been reported with a very low risk [3].

In Germany, testing of all blood donations for HEV is in preparation to be introduced. A nationwide HEV RNA universal screening of blood donations has already been introduced in Ireland, the UK, Japan and the Netherlands. In Switzerland, nucleic acid screening of all blood products for HEV started in November 2018 [4]. Blood authorities in Greece, Portugal, Italy, France and Spain are evaluating the situation [5]. Recently, the responsible German authority, the Paul-Ehrlich-Institut, recommended testing all blood donations for HEV. Here, we discuss the relative risk, posed by HEV transmission via transfusion and summarise the major arguments recommending the testing of all blood donations for HEV.

## HEV - biology and prevalence

HEV is a single-stranded, positive-sense RNA virus classified in the family *Hepeviridae* [6]. The HEV particles present in feces and bile are nonenveloped, while those in circulating blood and culture supernatants are covered with a cellular membrane, similar to enveloped viruses and called quasi-enveloped [7]. There are eight genotypes of HEV: HEV-1 and HEV-2 are primarily human viruses, whereas HEV-3 and HEV-4 were found in pigs, humans and other animals. HEV-5 and HEV-6 were described only in wild boars in Japan, HEV-7 was found in a dromedary camels and HEV-8 in Bactrian camels [8]. HEV-1 and HEV-2 are endemic in regions in Asia and Africa, the major source of infection is contaminated water (for review see [6]). HEV-3 and HEV-4 are zoonotic viruses transmitted to humans mainly by direct contact with pigs or eating undercooked pork. Seroprevalences of HEV-3 in domestic pigs were estimated between 5 and 100% [9]. The detection of viral RNA and virus-specific antibodies depends significantly on the analysed pig material, the age of the animals and the detection methods (PCR, real-time PCR, ELISA and Western blot analysis) [10–12].

Usually pigs are infected at an early age after the loss of the maternal antibodies, a peak of viral excretion in pig fecal samples is detected between week three and eight after weaning, the excretion decreases with the appearance of antibodies. Detection of viral RNA in serum samples is less frequent than in liver and fecal samples [9]. HEV-7 was also found in a liver transplanted patient regularly consuming camel meat and milk [13]. HEV infections have little impact on animal health, the animals have no obvious symptoms, however co-infection with other viruses may increase the amount and the duration of HEV secretion or induce even fatal diseases [14].

## Transmission of HEV-3 in pigs and humans

Whereas oral-fecal transmission is the major way of HEV transmission in pigs, transmission via the placenta was also detected [15]. Contact with pigs and the consumption of undercooked pork meat is the main way of transmission of HEV-3 to humans. HEV was also found in wild boars [16–18] and screening of wild boar hunters, forest workers and pet pig holders gave good evidence that HEV is transmitted by contact with pigs and wild boars [19, 20]. Many reports describe the detection of HEV RNA in pig liver, meat and meat products intended for human consumption (for review see [6]). In addition, HEV infections have been reported after consumption of shellfish and vegetables, obviously due to a contamination with pig manure [21–23]. Transmission has also been reported after consumption of wild boar meat [24].

The seroprevalence of HEV-3 in the general population of Western countries has been shown to differ, partially due to the use of different assays [10, 11, 25].

The HEV seroprevalence and viremia in blood donors was also shown to differ in Western countries (Table 1) [26–40]. In the Southwest of France even 52% positive donors were found, here mainly due to the consumption of undercooked *figatellu* liver sausages [27]. Studies in Germany showed an anti-HEV IgG prevalence of 6.8% [41], the rate of HEV-positive donations was 1:4525 [42] and hepatitis E viraemia was found at a relatively high rate of 0.12% among blood donors in 2018 [42]. In comparison with Europe and the United States, the seroprevalence was lower in Latin America [43, 44]. In Switzerland on average 29.4% of blood samples were positive, the seroprevalence increased with age (30.7% of males and 34.2% of women over 60 years old) [4]. An increase of seropositive donors with age was also observed in France [27].

**Table 1:** HEV seroprevalence and viremia in blood donors in Western countries

Country	HEV IgG positive (%)	RNA positive	Reference	Year of publication
Germany	29.5	1:1200	[26]	2012
France	22.4	1:2218	[27, 28]	2014, 2016
		1:744	[29]	2017
The Netherlands	27.0	1:2671	[30]	2012
		1.600	[31]	2015
England	12.0	1:2848	[32]	2011
		1:7000	[33]	2012
Scotland	4.7	1:14520	[34]	2013
Denmark		1:2330	[35]	2016
Spain	19.9	1:3333	[36]	2015

Due to the high prevalence in blood donors, transmission of HEV by blood transfusion has been observed [4, 26, 32, 33, 44–47], but not all patients receiving HEV-positive blood got infected [33, 44, 46]. The infectivity of HEV-contaminated components for transfusion was in one study 50% [48] and seems to depend upon the virus load [33, 46]. HEV transmission depends not only on the virus load, but also on the blood component given and on the presence of antibodies in the preparation [33]. Overall, donations containing antibody were less likely to transmit. HEV transmission was also associated with organ transplantation, but only in single cases [49–51]. The prevalence of persistent HEV infection in patients with solid organ transplantation in Western Europe varies between 0.7 and 3.2% [52]. The different observed incidences strongly depend on the respective antibody test applied in the respective study.

Most infections of immunocompetent humans with HEV-3 are asymptomatic, in rare cases a mild anicteric illness and a moderate hepatitis were reported. In some cases an acute hepatitis by red blood cell transfusion was described in immunocompetent subjects [53]. In a patient with thrombotic thrombocytopenic purpura (TTP), an acute HEV infection by cryosupernatant plasma triggered exacerbation of the TTP [54]. HEV infection becomes chronic in patients who are immunosuppressed (HIV-infection, transplantation, chemotherapy, rheumatic diseases), however chronic HEV infection was also observed in an immunocompetent patient with a history of systemic lupus erythematosus [55]. Neurological manifestations caused by HEV infections have been reported, but they are still not well analysed [56]. In patients with underlying liver disease, a severe hepatitis occurs [19]. HEV may be an immunosuppressive virus. There is evidence in a pig model of chronic HEV infection that HEV actively suppressed cellular immune responses [57] and increased levels of the immunosuppressive IL-10 have been found in HEV-infected individuals [58].

HEV infected individuals can be treated with the antiviral ribavirin and for prophylaxis, a human vaccine, at least against HEV-1, was developed. However the efficacy against HEV-3 has still not been specifically tested and this vaccine has no approval outside of China [59, 60]. There is evidence that the number of reported HEV-3 infections increases not only due to greater awareness and better testing, but also due to a real increase in infection rates [31, 36, 61]. However, in Switzerland the number of human infections declined [40]. Within the specified region of the Swiss Bern canton, overall prevalence has declined over two decades from 30.3% in 1997/98 to 27.0% in 2006 and 22.3% in 2015/6 [4].

## The need of testing of all blood donations

As mentioned above, several reports have described transfusion-transmitted HEV infections [4, 26, 32, 33, 42, 44, 46, 47]. In one case, HEV transmission was associated with fatal hepatitis in a Japanese cancer patient [62]. However, it is obvious that the risk of infection through meat consumption is much higher compared with transmission by blood transfusion. Screening blood donations will not prevent most cases of HEV transmission, but it will significantly reduce the risk for the transfusion recipients, which are either immunosuppressed and/or suffering from different diseases. In addition, there is a great difference between a person who takes up the virus orally and a person who gets infected intravenously. In the case of oral uptake there may be some protection by the acidic environment of the stomach and the mucosal barrier in the gut. Immunocompromised patients need often multiple transfusions, which increases the risk from transmission [46].

HEV was detected in 1 of 815 blood donations in Germany [45] and is 100 times more prevalent compared with HIV, HCV and HBV combined. Risk estimations considering both, the HEV prevalence and clinical risk for immunocompromised patients [63, 64] led to introduction of blood screening for HEV in the Netherlands and Germany. Analysing the cost-effectiveness of the screening of blood donations for HEV in the Netherlands, the authors came to the conclusion that preventing HEV transmission by screening of blood donations appears not excessively expensive compared to other blood screening measures [63]. However, since only a small number of HEV infections are due to blood transfusions, the overall impact on HEV disease burden will be small [63]. To reduce de novo infections in general, several strategies have been proposed [65].

It may be assumed that it may be sufficient to test only the blood donations for people at risk, e.g., immunosuppressed organ transplant recipients and recipients of haematopoietic stem cells. Such a limited testing seemed cost effective while protecting patients at major risk. However, there are several arguments for universal screening of all blood donations. First, the category “people at risk” is difficult to define due to individual variability. Cancer patients under chemotherapy, HIV infected individuals, and patients with rheumatoid arthritis and other rheumatic diseases under immunosuppressive treatment should also be included. The fatal outcome of a transfusion-transmitted infection of a cancer patient in Japan [62] underlines that the risk-group should not be limited to transplant patients alone. Second, even immunocompetent patients may suffer from acute HEV-infection or induced complications under circumstances requiring blood or plasma transfusion [54, 55, 66, 67]. Third, the handling of two separate types of blood donations would increase logistical cost and may even lead to product losses due to the stockpiling of tested and non-tested donations. For example, in the case more tested material is required, stored non-tested material will not be used and may expire and vice versa. Fourth, it may be very difficult or even impossible for a physician to assign a patient to an HEV risk in an emergency requiring transfusion.

## Conclusion

HEV-contaminated food is the main source of HEV transmission to humans in Europe and other industrialised countries with moderate climate conditions. However, transmission by blood transfusion can be dangerous for the recipients considering their immunosuppressive status, underlying disease or other circumstances requiring blood transfusion. Several of the arguments discussed above in detail argue in favour of testing all blood donations for HEV-3 to prevent transmission. Based on these arguments, blood donor testing has been introduced in several industrialised countries, including recently Germany.

## Data Availability

Not applicable.

## Abbreviations

ACLF: Acute-on-chronic liver failure
HBcAg: Hepatitis B core antigen
HBsAg: Hepatitis B surface antigen
HCV: Hepatitis C virus
HEV: Hepatitis E virus
HIV: Human immunodeficiency virus
HTLV: Human T cell lymphotropic virus
NAT: Nucleic acid testing
WNV: West Nile virus
